# Commentary: Spatiotemporal Modeling of the Key Migratory Events During the Initiation of Adaptive Immunity

**DOI:** 10.3389/fimmu.2019.02311

**Published:** 2019-09-24

**Authors:** Johannes U. Mayer

**Affiliations:** Malaghan Institute of Medical Research, Wellington, New Zealand

**Keywords:** cell migration, dendritic cells, adaptive immunity, innate immunity, cell tracking, Kaede mice, photoconversion

The priming of adaptive immune responses in the draining lymph node is a crucial step to initiate functional T and B cell responses against newly encountered antigens present in the periphery. While lymph node resident antigen-presenting cells have been reported to present free draining antigen that is transported by lymph and accumulates within the subcapsular sinus shortly after immunization (1–3), the majority of cells that migrate from the tissue to the lymph node arrive after 24 h. A large proportion consists of different populations of conventional dendritic cells, which migrate through the lymphatics to transport antigen from the periphery to the lymph node where they present antigen peptides to naïve T cells to initiate adaptive immunity [recently reviewed in Worbs et al. (4) and Randolph et al. (5)]. However, antigen positive neutrophils, monocyte derived dendritic cells, and monocytes have also been identified in the lymph node under certain conditions (6–9), but defining their migration kinetics and origin remains challenging.

To gain a better understanding of the key migratory events that are elicited by Alum/LPS, a model for Alum-based vaccine administration, Hayes et al. performed a detailed analysis of the cell migration kinetics and antigen presentation events in the draining lymph nodes after footpad immunization (10). Only a limited number of tools are available to monitor the dynamic migration events that occur during an immune response *in vivo*, with each tool presenting their own advantages, challenges, and limitations.

While two-photon microscopy allows for *in vivo* imaging in real time, it requires a challenging experimental setup when inner tissues are studied and limits the observation to the fluorescent cell types that can be visualized using reporter mouse strains (11, 12).

A more unbiased approach is achieved by cannulating the lymphatic vessels and collecting lymph *ex vivo*. While small amounts of lymph can be collected via a glass capillary from many locations we can only gain insights into its temporal composition (11). Access to large lymphatic vessels is necessary for the cannulation and continuous collection of lymph. This procedure has been successfully performed for thoracic duct cannulations in mice and rats and allows for the direct assessment of lymph migratory cell populations at steady state and after intestinal immunization (12–14). However, migrating cell populations from other tissues are harder to assess as the smaller diameter and sequential positioning of lymph nodes along smaller lymphatic vessels makes the insertion of a cannula at the right location challenging (15).

With the development of photoconvertible fluorescent proteins (namely mEosFP, tdEosFP, Dendra, Dronpa, Kaede, KikGR, and mOrange) and the availability of transgenic mouse strains that either ubiquitously or selectively express these proteins in all cells or certain cell types or organelles (16), tissues of interest can now be photoconverted and migrating cell populations tracked to non-converted tissues.

To conduct a successful photoconversion experiment *in vivo*, several important details need to be considered:

- An appropriate light source needs to be chosen that emits the optimal wavelength to photoconvert the fluorescent protein, allows for deep penetration into the tissue to ensure complete photoconversion, and does not cause damage, cell activation or inflammatory reactions that could cause unspecific cell migration.
- The selection of the experimental model and the cell types studied need to ensure that a distinct photoconverted signature can be detected when cells are collected and that the fluorescent signal is not diluted due to cellular degradation of the photoconverted protein or excessive cell division.
- The timing of the photoconversion needs to take into account that only cells present at this specific timepoint will be photoconverted, while cells that have already migrated away or subsequently arrive in the tissue will not be distinguishable from non-migrating cells.

When these parameters are appropriately selected, photoconvertible mouse strains are an easy-to-use and flexible option to study the migration kinetics of immune cells at steady state or in response to immunization.

Using ubiquitously expressing Kaede mice, the migration of dendritic cell subsets from the skin and intestine has been well-characterized and were found to be the only tissue migrating immune cell that migrates from the tissue to the lymph node via the lymphatics under steady state conditions (17–19). To study cellular migration during an immune response a large variety of models have been used that range from inducing unspecific inflammation through tape-stripping, the epicutaneous application of irritants or cell labeling dyes, to the subcutaneous or intradermal injection of dyes, adjuvanted proteins, nanoparticles, or pathogens.

To better define the phenotype and kinetics of skin migrating cells after Alum/LPS treatment, Hayes et al. treated photoconvertible Kaede mice with Alum/LPS and tracked the photoconverted cells from the site of treatment to the draining lymph node (10). By combining data from lymph nodes collected at different time points after photoconversion and immunization and modeling the resulting cell migration kinetics mathematically, the authors conclude that migratory cell populations first accumulate in the footpad after Alum/LPS treatment, migrate to the lymph node at a fixed rate and remain in the lymph node for a prolonged period of time compared to the administration of saline (10). While previous studies show that migrating dendritic cells are first detected 3–8 h after egress from the skin (20, 21), Hayes et al. show here that the majority of dendritic cells that accumulate in the lymph node after Alum/LPS treatment accumulate in the tissue 8–12 h after immunization and migrate to the lymph node at a peak time of 24–36 h (10). In contrast to other models that use tape stripping or deliver non-adjuvanted antigens by injection (13, 20, 22), the current study further indicates that the majority of migrating immune cells are not already present in the tissue, but first need to be recruited to the skin after Alum/LPS treatment (10).

While it is assumed that depot formation and the slow release of antigen or inflammatory mediators are responsible for the adjuvanticity of alum (and could explain the accumulation of immune cells), the same laboratory has shown that both alum and CpG induced similar uptake of antigen in the lymph node, regardless of depot formation (23). This suggests that the majority of migrating cells that migrate after Alum/LPS treatment are recruited from outside the tissue and poses the question if the identified population of dendritic cells are indeed conventional tissue-resident dendritic cells. Alternatively, monocyte derived dendritic cells, which accumulate at the site of immunization, have been reported after alum treatment (9), and can express similar levels of MHCII and CD11c after activation (24) could have been reported in this study. It therefore remains to be determined if the migrating dendritic cells observed here are monocyte derived dendritic cells (10), or if they represent conventional dendritic cells that have developed from rapidly arriving dendritic cell progenitors as recently reported for viral infections (25). If identified as monocyte derived dendritic cells, this study would be the first to provide formal evidence that monocyte derived dendritic cells can migrate from the inflamed tissue to the draining lymph nodes via the lymphatics, and do not enter the lymph node via the bloodstream, as currently believed (26).

This would suggest that a therapeutic intervention or targeting of migrating cell populations after Alum/LPS treatment or Alum-based vaccinations should be delayed to account for their accumulation in the tissue and could be developed as an effective tool to influence the outcome of adaptive immune responses in the lymph node.

## Author Contributions

The author confirms being the sole contributor of this work and has approved it for publication.

### Conflict of Interest

The author declares that the research was conducted in the absence of any commercial or financial relationships that could be construed as a potential conflict of interest.
